# Effects of intrinsic-foot-muscle exercise combined with the lower extremity resistance training on postural stability in older adults with fall risk: study protocol for a randomised controlled trial

**DOI:** 10.1186/s13063-021-05554-5

**Published:** 2021-09-03

**Authors:** Zhangqi Lai, Hongbo Pang, Xiaoyue Hu, Kun Dong, Lin Wang

**Affiliations:** 1 School of Kinesiology, Shanghai University of Sport, Shanghai, China; 2 Tianjin Rehabilitation guidance Center for the Disabledg, Tianjin, China

**Keywords:** Postural stability, Randomised controlled trial, Foot core training, Fall, Intrinsic-foot-muscle exercise

## Abstract

**Background:**

Falls are one of the most common accidents in older adults, often leading to injury, disability and quality-of-life declines. Foot core function contributes to postural stability in most static postures and dynamic activities. As efficient foot core training, the intrinsic-foot-muscle exercise has been proposed to improve postural control. However, the effects of the exercise on postural stability in the elderly remain unclear. Therefore, this study attempts to investigate the effect of 12-week intrinsic-foot-muscle exercise on postural stability in older adults with fall risk.

**Methods:**

We will conduct a prospective, single-blind randomised controlled trail on 120 older adults with fall risk. Participants will be randomly assigned to an intrinsic-foot-muscle exercise combining the lower extremity resistance training group (IFM group), an extrinsic-foot-muscle exercise combining the lower extremity resistance training group (EFM group) and a control group. The control group will perform lower extremity resistance training. The IFM and EFM groups will be given additional short-foot exercise or towel-curl exercise training, respectively. After the intervention, participants will be followed up for another 12 weeks with no active intervention. The outcome measures will include the postural stability measurements, self-reported postural stability, number of falls, intrinsic-foot-muscle strength and foot arch function. Furthermore, adverse events will be recorded and analysed. If any participant withdraws from the trial, an intention-to-treat analysis will be performed.

**Discussion:**

The trial is designed to investigate the efficacy of a 12-week intrinsic foot muscle training combined with the lower extremity resistance training on postural stability outcomes in elderly people with fall risk. The trial will also examine the comprehensive outcomes of postural stability during static standing and dynamic movements. The function of intrinsic foot muscle to support the arch will also be evaluated. Important features of this trial mainly include intervention setting, outcome measure selection and study duration. The results of this study will determine the effectiveness and provide scientific evidence to establish comprehensive fall prevention intervention.

**Trial registration:**

Chinese Clinical Trial Registry ChiCTR2000033623. Registered on 7 June 2020. http://www.chictr.org.cn/showproj.aspx?proj=54741

## Administrative information

Note: the numbers in curly brackets in this protocol refer to SPIRIT checklist item numbers. The order of the items has been modified to group similar items (see http://www.equator-network.org/reporting-guidelines/spirit-2013-statement-defining-standard-protocol-items-for-clinical-trials/).

**Table:** 

Title {1}	Effects of intrinsic-foot-muscle exercise combined with the lower extremity resistance training on postural stability in older adults with fall risk: study protocol for a randomized controlled trial
Trial registration {2a and 2b}.	Chinese Clinical Trial Registry, ID: ChiCTR2000033623. This study has been registered on 7 June 2020.
Protocol version {3}	Protocol version number 1, dated July 04, 2020.
Funding {4}	The National Key Technology Research and Development Program of the Ministry of Science and Technology of China (2019YFF0302101).
Author details {5a}	Zhangqi Lai ^1^, Hongbo Pang ^2^, Xiaoyue Hu^1^, Kun Dong^1^, Lin Wang ^1^ 1 School of Kinesiology, Shanghai University of Sport, Shanghai, China 2 Tianjin Rehabilitation guidance Center for the Disabled, Tianjin, China
Name and contact information for the trial sponsor {5b}	Not applicable. There is no sponsor for this study.
Role of sponsor {5c}	The funder has no input in the study design, protocol preparation, or future data analysis and interpretation.

## Introduction

### Background and rationale {6a}

Falls are the second leading cause of unintentional injury-related deaths among older adults, and falls can cause nonfatal injuries such as fracture and head injury [1, 2]. According to data of the Centres for Disease Control and Prevention, approximately one-third of people aged above 65 years in the USA reported falling in the preceding 12 months [3], and half of those fallers will fall again within the subsequent year [4]. Furthermore, falls substantially reduce the mobility and balance confidence of older people, imposing a significant medical and social burden. As estimated, the medical costs caused by fatal and nonfatal falls were approximately 50.0 billion USD for 1 year in the USA [5]. Similarly, in China, the aging and increasing fall-related injury in older adults is a growing public health concern. The medical cost of fall-related injury among older Chinese people ranged from 16 to 3812 USD per person per fall [6].

Falls, as a major public health issue, have attracted the attention of many researchers. For older individuals, multiple factors and complications are associated with falls, such as obesity, low lower-limb muscle quality and impaired postural control [7, 8]. Postural control is among the most common causes of falls in older adults, often leading to injury, disability and quality of life declines [9]. The functions of the vision, vestibular and musculoskeletal systems, as well as cognitive function, play an important role in human postural stability [10–12]. These physiological functions related to posture control would decline with age. Thus, effective intervention strategies to improve postural stability in the elderly are urgently needed.

The human foot is a highly complex structure, and it contributes to postural stability in most static postures and dynamic activities [13]. Owing to the technical constraints, previous researches of postural control focused on related physiological factors and postural adjustment strategies, while ignoring the role of the foot as an independent structure in postural control [7, 14]. In 2015, McKeon et al. [15] proposed a new paradigm of the foot core system that further refined the foot structure into active, passive and neural subsystems. As a direct interface between the body and the ground, the stability of the foot arch (passive subsystem) is a requisite to normal foot function and considered the core function of the foot [15]. Based on the coordination between controls of muscular activity (active subsystem) and the somatosensory afferent system (neural subsystem), the feet help to sense and interact with the environment [16].

Recently, with the development of foot and ankle biomechanics, several researchers have started to explore the role and mechanism of the foot core system in human postural stability [17, 18]. Previous studies suggested that the weakness of foot core stability is related to the decline of postural stability [19–21]. The functions of foot arch (passive subsystem) and foot sensory (neural subsystem) were associated with postural stability, and they declined with age [22, 23]. The intrinsic foot muscles and extrinsic foot muscles, which constitute the foot active subsystem, also play an active role in maintaining foot core stability [15]. Several studies have speculated that local dynamic support of the foot arch was provided from the intrinsic foot muscles [24].

Owing to a lack of understanding of intrinsic foot muscles, limited studies were designed to investigate the effect of the intrinsic muscles on postural control. Lee et al. [25] found that 8-week intrinsic foot training was more effective to improve the proprioception, dynamic balance and ankle stability index in individuals with ankle instability than traditional proprioceptive sensory training. In another study, after a 4-week programme of intrinsic foot training, a significant decrease was reported in medio-lateral displacement of the centre of pressure (COP) during the dynamic balance test [26] indicating the improvement in postural stability during dynamic activity. Studies have mainly focused on the postural control of healthy young adults and ankle instability, rather than individuals with balance disorders, such as the elderly. The effect of foot core stability training on the static postural control and the dynamic postural stability with the elderly must be investigated to develop a multifactorial effective management for the older adults with fall risk.

### Objectives {7}

Accordingly, the present study attempts to investigate the effects of intrinsic foot muscle intervention on the static postural control and the dynamic postural stability of older adults with fall risk. We will conduct a single-blind randomised controlled trial to investigate the efficacy of a 12-week intrinsic foot muscle training programme combined with the lower extremity resistance training on comprehensive outcomes in individuals with fall risk. The results of this study will determine the effectiveness and provide scientific evidence to establish comprehensive fall prevention intervention.

### Trial design {8}

**Fig. 1:**
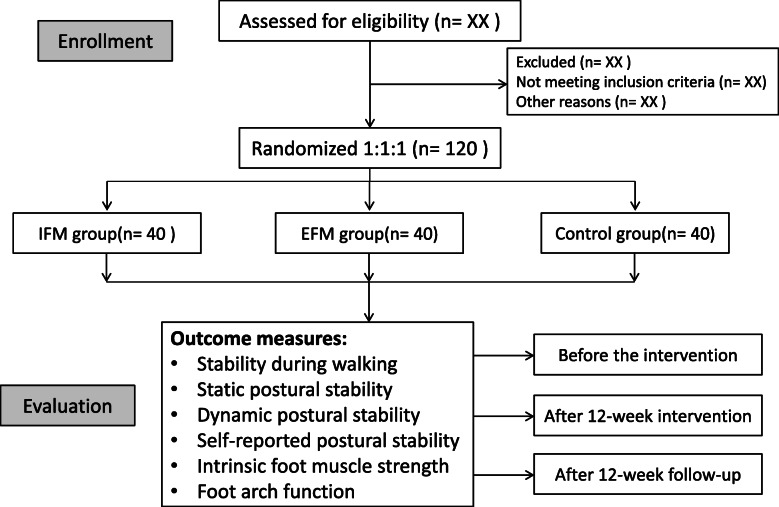
Flow diagram of study design. IFM group, intrinsic-foot-muscle exercise combined the lower extremity resistance training group; EFM group, extrinsic-foot-muscle exercise combined the lower extremity resistance training group; Control group, lower extremity resistance training group

This is a protocol for a parallel-group, single-blind randomised controlled trial (Fig. 1). It will follow the recommendation and suggestion of CONSORT 2010 (Consolidated Standards of Reporting Trials). The study will be carried out over a 12-week intervention (i.e., lower extremity resistance training), delivery and a 12-week follow-up.

Eligible participants will be randomised at a 1:1:1 ratio to enrol in one of the three groups. Three intervention programmes will be included in this study, namely, intrinsic-foot-muscle exercise combined with lower extremity resistance training (IFM), extrinsic-foot-muscle exercise combined lower extremity resistance training (EFM) and lower extremity resistance training programme (Fig. 1).

## Methods: participants, interventions and outcomes

### Study setting {9}

This study will be conducted at the Sport Medicine and Rehabilitation Centre, Shanghai University of Sport. In this study, the older participants with fall risk are defined by fall history and function test. The participants will be recruited from several community centres in Yangpu District, Shanghai, China. Before the initiation of the study, all participants will offer their basic information, including the medical history, history of falls and exercise habits. All personal data will be confidential and each subject will be provided informed consent before the inclusion in the study.

### Eligibility criteria {10}

Before baseline measurement, all participants will undergo screening to determine their current fall risk, including the self-report fall history for past 6 months and the Timed Up and Go (TUG) assessment [27].

#### Inclusion criteria

1. Adults older than 60 years old
2. Able to maintain the standing position
3. Able to walk alone, without others, prosthesis or mobility aids
4. Normal cognitive function
5. Be at moderate fall risk which is determined by one fall within the last 6 months and more than 10s of the TUG test [27]
6. Availability: three times a week over 12 weeks

#### Exclusion criteria

Exclusion criteria include the following:

1. Participants diagnosed with diseases related to postural control, such as vestibular dysfunction, Alzheimer’s disease, Parkinson’s disease and motor neuron disorders
2. History of lower limb trauma in the past year
3. Severe cardiopulmonary disease
4. Participants with medical contraindications for exercise
5. Currently undertaking a structured exercise programme for postural control

### Who will take informed consent? {26a}

Those who satisfy the inclusion criteria and willing to participate this experiment will be contacted by the same investigator (ZL) to be informed of the procedures of this study and to arrange the baseline assessment of outcomes. Each subject will be informed of the benefits and possible risks, and they will sign informed consent before their inclusion in the study which explains the study in detail about purpose, procedures, potential risks, benefits and confidentiality of the information.

### Additional consent provisions for collection and use of participant data and biological specimens {26b}

No biological samples will be obtained to be stored for use in this study. Additionally, the participants will be asked for permission of the continued use of their data in case of withdrawal from the trial or relevant studies in the future.

### Assignment of interventions: allocation

#### Sequence generation {16a}

The participants will be estimated by the Berg Balance Scale. Score ranging from 45 to 56 points indicates low fall risk, and score ranging from 21 to 40 points indicates medium fall risk, while score ranging from 0 to 20 points indicates high fall risk. They will be stratified based on the Berg Balance Scale and be randomised allocated to three groups. A randomisation sequence will be created by the main investigator (LW). And the randomisation will be carried out in blocks by computer-generated randomisation.

#### Concealment mechanism {16b}

The group randomisation will be separated into opaque envelopes kept by the main investigator. In addition, each participant will be represented by a separate number.

#### Implementation {16c}

The procedure of allocation sequence will be conducted by an independent researcher (the main investigator, WL). The investigator (ZL) will take charge of the participant recruitment (informing them the trail procedures and obtaining their informed consent before inclusion in the study). Furthermore, another investigator (HP) will assign participants to interventions.

### Assignment of interventions: blinding

#### Who will be blinded {17a}

One physical therapist in charge of interventions will be blinded for baseline assessment. Furthermore, the researchers responsible for data collection and data analysis will be blinded for the random allocation. The participants will be instructed not to divulge the intervention accepted.

#### Procedure for unblinding if needed {17b}

Unblinding is permissible only when a serious adverse event or emergency rescue occurs.

### Interventions

#### Explanation for the choice of comparators {6b}

Although the optimal features of successful fall prevention exercise programmes are not yet clear, it has been recommended that the exercise aiming at improving lower extremity strength should be included in effective fall prevention programme. Therefore, the lower extremity resistance training was to be selected as a comparative intervention [28–30]. In this study, participants in IFM and EFM groups will extra receive two different types of foot muscle exercise, namely, short-foot exercise and towel-curl exercise. As a common foot muscle strengthening exercise, the towel-curl exercise primarily recruits extrinsic foot musculature, whereas the short-foot exercise has gained popularity involving isolate activation of the intrinsic foot musculature to reinforce the foot arch effectively [24, 26]. Different from the towel-curl exercise, during the short-foot exercise, the foot will be shortened by drawing the first metatarsophalangeal joint towards the calcaneus without toes curling [15].

#### Intervention description {11a}

The participants in each group will participate in respective intervention programme for 12 weeks. All training sessions will be done by a qualified physical therapist.

##### Intrinsic-foot-muscle exercise combined the lower extremity resistance training group (IFM)

Participants in the IFM group will be instructed to perform short-foot exercise according to the methods used in a previous study [25]. Under the supervision of the physical therapist, they will shorten their feet in the anterior-posterior direction without curling the toes, by actively drawing the head of the first metatarsal towards the heel. In short-foot exercise, the foot should be held for 5 s per repetition. The intensity of exercise will be divided into three levels (sitting position, double-leg stance and single-leg stance) with the overload increasing progressively. In general, for weeks 1–4, the IFM will be performed in a sitting position, subsequently performed in double-leg standing for weeks 5–8, and finally performed in single-leg standing for weeks 9–12.

Meanwhile, participants in the IFM group will undertake lower extremity resistance training. Thera-Band® resistance bands (The Hygenic Corporation, Akron. OH, USA) will be used during strength training. Likewise, training sessions will be supervised by a physical therapist to ensure correct training method and provide security. The programme of lower extremity resistance training includes extended leg raises, hip abduction, hip adduction, hip extension, knee extension, knee flexion, ankle plantarflexion and ankle dorsiflexion. The training will be progressed by increasing the strength of resistance bands to provide greater resistance.

The exercise regimen will comprise 3 blocks of 12 repetitions for IFM and resistance training, with a 2-min rest period between blocks. Each set will be performed bilaterally. Three sets will be performed 3 times per week for 12 weeks.

##### Extrinsic-foot-muscle exercise combined the lower extremity resistance training group (EFM)

The EFM group will undertake the towel-curl exercise and lower extremity resistance training group. During the towel-curl exercise, the physical therapist will place a towel on the floor and direct participants to place toes on the edge of the towel. Then, the participants will be instructed to drag the towel by flexing their toes. Similarly, the grip on the fabric will be held for 5 s per repetition [26]. Participants will perform the EFM exercise progressively under the position of sitting position, double-leg stance and single-leg stance like the IFM. The resistance training in the EFM group will be the same as the protocol in the IFM group.

The exercise regimen will be consistent in each training group. The participants will perform this training three times per week for 12 weeks.

##### Control group

The participants in the control group will perform the same lower extremity resistance training as the IFM and EFM groups. The training will also be supervised by the physical therapist. The entire intervention will be conducted under the protocol of the IFM and EFM groups and for the same duration.

#### Follow-up after intervention

The three groups will follow their respective training programmes for 12 weeks and will be followed up for another 12 weeks. After the intervention period, the participants will undergo a 12-week training period at home. During follow-up, they will be asked to maintain their previous lifestyle and continue interventions, respectively, and the physical therapist will provide guidance by telephone approximately once every 2 weeks. At week 24, these participants will return the laboratory and be reassessed.

### Outcomes {12}

This study will include assessments at the following time points: before intervention, after 12 weeks of intervention and after 12 weeks of further follow-up with home-based interventions. The total study period will be 6 months. All outcome measures assessments will be conducted by research assistants who are blinded to the subject assignment. Initially, all the participants will be submitted to fall risk test, comprised by the self-report fall history for the past 6 months and the TUG assessment. In addition, demographic data will be collected before the intervention. The data include subject characteristics (i.e., sex, age, height, weight, body mass index, degree of education and current drug treatment). Mentioned above tests will be conducted in a controlled environment by a trained researcher.

#### Primary outcome measures

##### Static postural stability

The NeuroCom Balance Manager System (Version 9.3, Copyright ©1989–2016 Natus Medical Incorporated) will be used to determine static postural stability. The balance platform is equipped with 18×60″ dual-force plate to measure the changes in COP of each participant. The COP will be collected when the participants stand on both feet with eyes open or eyes closed. We will calculate the same parameters as the postural stability test during standing: total length (TL), sway area (SA), maximum range of sway in the diction of AP, maximum range of sway in the diction of ML, mean velocity of COP sway and coefficient of variance of COP sway. The outcome data of static postural stability will be formatted as the difference at the points of baseline (week 0), post-intervention (week 12) and follow-up (week 24). The values will be shown as mean and standard deviation.

#### Secondary outcome measures

##### Stability during walking

In this study, the insole plantar pressure distribution measurement system (Novel Pedar, Germany) will be used to determine postural stability during the walking. Each insole contains 99 pressure sensors, which help to collect plantar pressure accurately and sensitively and to calculate the changes in COP in different tasks [31]. In advance of the testing, the pressure insole that fits the size of the participant’s shoe will be inserted into the left and right shoe and sampled at 100 Hz. Both insoles will be calibrated before data collection. The pressure measurement system will be presented to each participant and all procedures will be explained.

During the testing, the participants will be asked to walk 10 steps on a horizontal trail at their accustomed walking speed. Uniform shoes will be provided to prevent the effect of differences in shoes of the various participants. Three successful tests will be recorded for analysis without any unexpected movements in walking. To avoid the effects of walking start and braking, the data of the middle five steps of walking will be used for analysis. After we set the medial-lateral direction (ML) as *X*-axis and the anterior-posterior direction (AP) as *Y*-axis, the system will calculate the COP of each foot and it will be extracted to COP_L_ (*x*_1_, *y*_1_) and COP_R_ (*x*_2_, *y*_2_). Accordingly, the single COP for the whole body will be determined as ($\frac{x1+x2}{2}$, $\frac{y1+y2}{2}$). Then, we will calculate the following features from COP: TL, SA, maximum range of sway in the diction of AP, maximum range of sway in the diction of ML, mean velocity of COP sway and coefficient of variance of COP sway. The outcome data of stability during walking will be formatted as the difference at the points of baseline (week 0), post-intervention (week 12) and follow-up (week 24). The values will be shown as mean and standard deviation.

##### Dynamic postural stability

To develop the study, the NeuroCom Balance Manager System (Version 9.3, Copyright ©1989–2016 Natus Medical Incorporated) will be recruited to determine postural stability by measuring the participants’ centre of gravity (COG) alignment at a sampling frequency of 100 Hz. The balance platform is equipped with 18×60″ dual-force plate to measure the vertical forces exerted through the participant’s feet. We will conduct the sensory organisation test (SOT) and limits of stability test (LOS) to evaluate the static postural stability. Similarly, the participants will be informed of all procedures in advance. They will be positioned with standardised foot placement relative to individual height and be instructed to stand facing forward, with their arms relaxed at their sides and to remain as still as possible. The participants will remove all footwear and be restricted by a seat belt.

For the SOT, the participants will perform all balance trials and repeat each trial three times [32]. The movements of COP will be recorded with the participants standing under six conditions (as shown in Table 1). The duration of running each condition was 20 s. In all trials, the assessor will not alert the participants to impending changes of surrounding or the platform, but the assessor will only instruct them to open or close their eyes. Each participant will complete these six trials in the random order.

**Table 1:** Six conditions in sensory organisation testing

Test condition	Eyes	Surroundings	Platform
1	Open	Fixed	Fixed
2	Closed	Fixed	Fixed
3	Open	Sway referenced	Fixed
4	Open	Fixed	Sway referenced
5	Closed	Fixed	Sway referenced
6	Open	Sway referenced	Sway referenced

By the combination of vision, surrounding and contact surface, the SOT test creates sensory-conflict situations and helps to evaluate the capacity to maintain postural stability under these sensory conflicts. According to the sway and sway velocity of COP, the system will generate equilibrium scores of each condition. An overall composite equilibrium score will be calculated using a weighted average of these scores and be used for analysis. A high equilibrium score means a low postural sway, indicating greater postural stability [33].

**Fig. 2:**
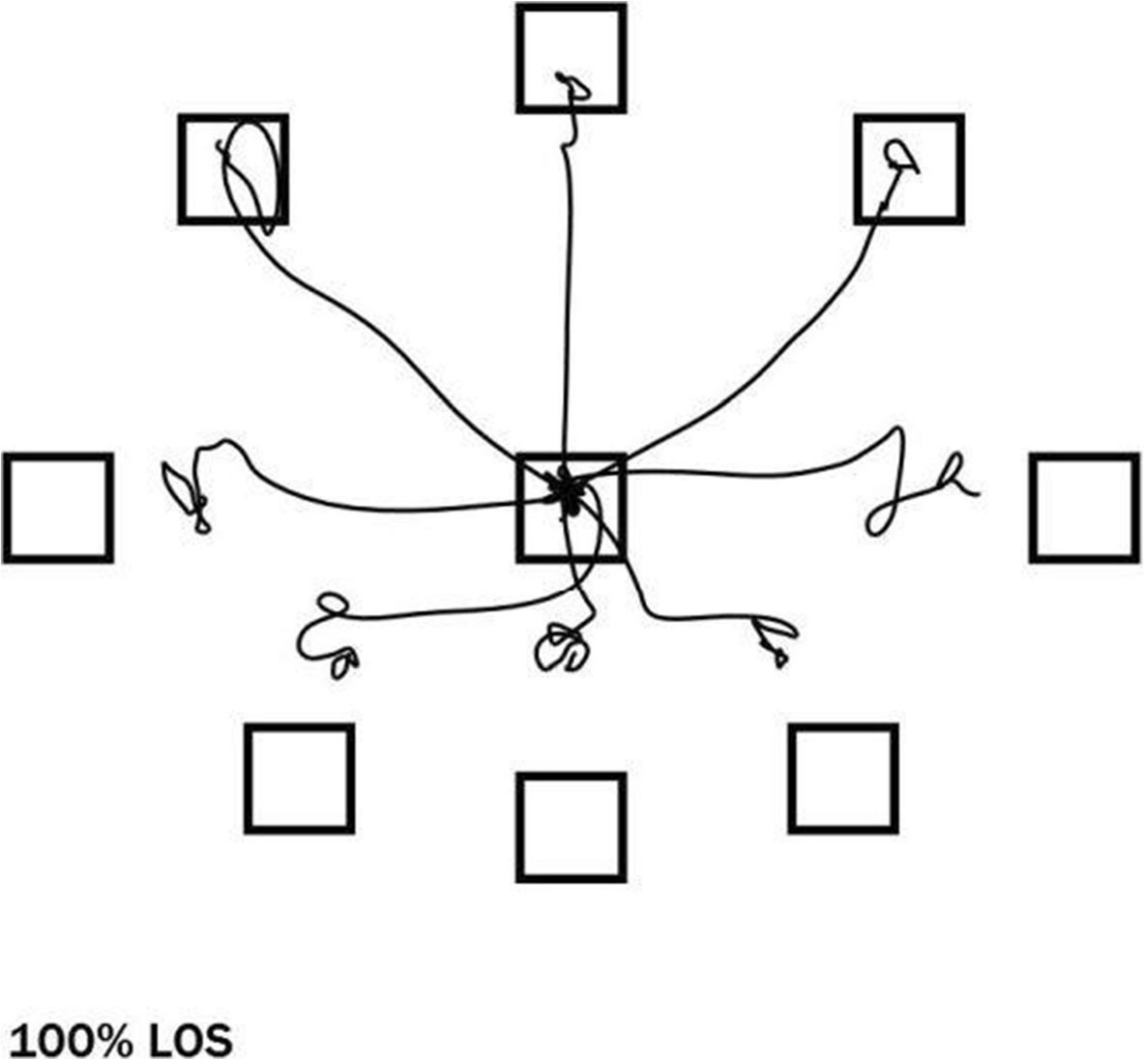
Limits of stability test

Furthermore, the LOS test will be conducted to measure the maximum distance by which a participant can intentionally displace COG [34]. The same standing position will be instructed as in the SOT test. In a different step, the computer screen at eye level in front of the participant will be turned on, depicting a centre box with eight target boxes equally spaced in an elliptical arrangement around the centre (as shown in Fig. 2). Participants will be instructed to shift their weight so that an icon (the projection of their COP) is held steady in the centre box. After the tone, they will lean their body to move the icon in these eight directions without losing balance, lifting their feet, or reaching for assistance. They will hold the icon steady in the target box until the second tone and return to the centre box. The test will begin with the target in a forward position and move in a clockwise direction.

The performance for trials in each of the eight directions will be recorded and used to calculate the following indicators. First is directional control, or the amount of movement in the intended direction minus the amount of extraneous movement, expressed as a percentage. Second is end-point excursion, or the distance travelled by the COG on the primary attempt to reach a target, expressed in percentage. Third is maximal excursion, or the furthest distance travelled by the COG. Fourth is movement velocity (average speed of the COG shift towards the target, measured in degrees per second).

The outcome data of dynamic postural stability (SOT and LOS tests) will be formatted as the scores at the points of baseline (week 0), post-intervention (week 12) and follow-up (week 24). The values will be shown as mean and standard deviation.

##### Self-reported postural stability

The Berg Balance Scale will be used to evaluate the postural stability of individuals in daily activities, which is widely used in scientific research for the elderly or patients with balance dysfunction [35]. It includes 14 questions scored from 0 to 4. The participants will rate according to the degree of difficulty. The outcome of Berg scale will be recorded at the points of baseline (week 0), post-intervention (week 12) and follow-up (week 24). The scores of Berg scale will be shown as proportion.

##### Self-reported number of falls

In addition, the number of falls in the last 3 months will be recorded by each participant. According to 12-week period of intervention and follow-up, the participants will be asked to recall the falls in the last three months in order to determine the treatment effect of these intervention. Similarly, the number of falls will be reported by participants at the points of baseline (week 0), post-intervention (week 12) and follow-up (week 24). The scores of berg scale will be shown as proportion.

##### Intrinsic foot muscle strength

To measure the strength of supporting the foot arch, the intrinsic foot muscle strength will be collected during a special functional movement known as ‘doming’. The measurement of intrinsic foot muscle strength will be conducted on custom-built apparatuses with a dynamometer, which has been reported in previous studies (ICC = 0.949, SEM = 0.883 kg) [36, 37]. The participants will be instructed to perform the doming movement by activating muscles to pull the metatarsal heads towards the heel (also known as the short-foot exercise). Before the test, the assessor will check the foot placement to ensure that the dynamometer is rested against the dorsum of the foot, just above the navicular tuberosity. Participants will be asked to perform the doming to a 3-s maximal voluntary contraction against the dynamometer. Only the data of correct movement will be recorded. The mean values of the three trials will be used for analysis. The value of foot muscle strength will be measured at the points of baseline (week 0), post-intervention (week 12) and follow-up (week 24). The scores of berg scale will be shown as mean and standard deviation.

##### Foot arch function

The navicular drop test, which have been used in older adults [38], is one clinical examination commonly used to evaluate foot arch function [39, 40]. It will be conducted to determine the effect of intrinsic-foot-muscle exercise on supporting the foot arch. First, with the participants standing barefoot on the floor, an experienced investigator will mark the navicular tuberosity. With the palpation of the lateral and medial aspect of the talar dome, the investigator will invert and evert the foot carefully until the talus is in a central position. The distance between the navicular tuberosity and the floor will be measured with a vernier calliper. Then, the investigator will repeat the test process while the participants stand in a non-weightbearing position. The difference in the navicular tuberosity heights will be calculated for analysis. The outcome of the navicular drop test will be measured at the points of baseline (week 0), post-intervention (week 12) and follow-up (week 24). The scores of berg scale will be shown as mean and standard deviation.

### Criteria for discontinuing or modifying allocated interventions {11b}

Withdrawal from the study will be allowed on the following:

1. The subject makes such a request.
2. The subject develops a serious disease, such as heart disease or stroke. He or she is unsuitable to continue the participation after the determination of the main investigator.
3. The subject reports serious adverse events in this study. Serious adverse events refer to the unexpected events that may require medical or surgical intervention, such as musculoskeletal problems.

### Strategies to improve adherence to interventions {11c}

The following measures will be taken to improve adherence to interventions and follow-up.

1. All participants will be informed earnestly of the testing arrangement, intervention routine, potential benefits and risks to make them fully understand the significance of their involvement in the study.
2. All participants will perform the intervention routine under the supervision of a qualified physical therapist.
3. The investigator (HP) will randomise participants to interventions and stay in touch with the participants.
4. Additionally, they will be coordinated duly to return to the laboratory for the measurement of post-intervention and follow-up.

### Relevant concomitant care permitted or prohibited during the trial {11d}

During the trial, the participants will be informed not to take part in any other regular balanced promotion programmes and maintain their previous lifestyle.

### Provisions for post-trial care {30}

Not applicable. There is no anticipated harm and compensation for trial participation.

### Participant timeline {13}

**Fig. 3:**
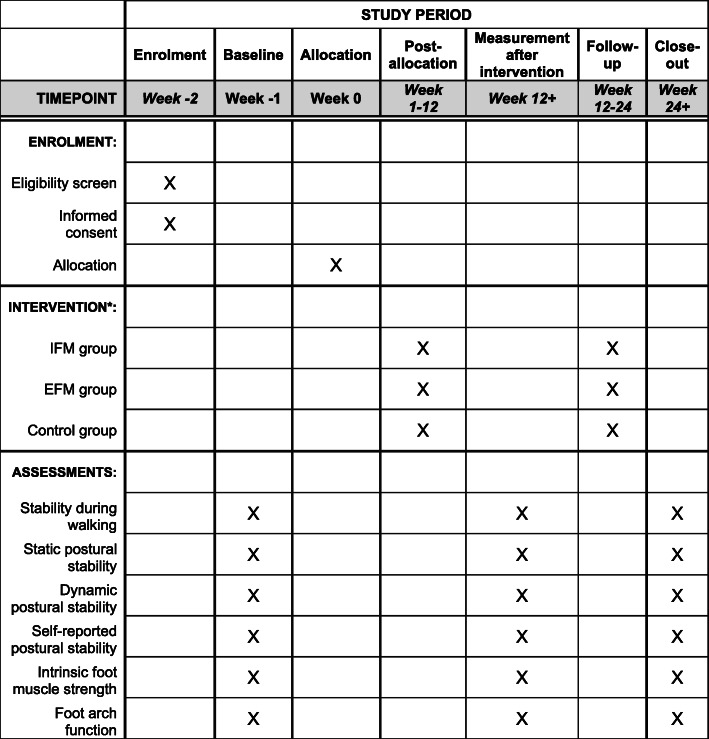
Schedule of study procedures. *IFM group, intrinsic-foot-muscle exercise combined the lower extremity resistance training group; EFM group, extrinsic-foot-muscle exercise combined the lower extremity resistance training group; Control group, lower extremity resistance training group

Figure 3 shows the time schedule of enrolment, visits for participants, intervention and assessments.

#### Sample size {14}

To the best of our knowledge, no study has been designed to investigate the effect of intrinsic-foot-muscle training on postural stability in older adults. Therefore, the G*POWER software 3.1 software was used to calculate the suitable sample size for the multivariate of analysis for repeated measurements (MANOVA) test using 3 independent groups with settings of *α* = 0.05, power (1 − β) = 0.80 and standardised medium effect size = 0.25 (the partial eta squared). The power analysis showed that three independent groups with 98 participants in total were the required sample size. Considering the drop-out rate, 40 participants in each group will be recruited for this study.

#### Recruitment {15}

The participants will be recruited through advertisements from several community centres in Shanghai, China. All participants will be screened by the research team (clinicians, physical therapist and exercise specialists) for participation based on the inclusion and exclusion criteria listed above. Those who satisfy the inclusion criteria and are willing to participate in this experiment will be provided details about study treatment, follow-up and contact details for further information. Each participant will be informed of the benefits and possible risks, and they will sign the informed consent and an ethics committee-approved personal information sheet before their inclusion in the study. Recruitment will begin in October 2020.

### Data collection and management

#### Plans for assessment and collection of outcomes {18a}

Before the trial, all researchers responsible for data collection will be trained to be proficient in test procedures and operations. The questionnaires and laboratory tests used in this study have been widely used in previous studies, and reliability and validity have been reported in the study. Outcome measurements and data collections will be conducted at the baseline, post-intervention (12 weeks) and follow-up (24 weeks).

#### Plans to promote participant retention and complete follow-up {18b}

During the 12-week follow-up, participants will be asked to maintain their previous lifestyle and continue their interventions respectively. The physical therapist will provide guidance by telephone weekly. At week 24, the participants will return to the laboratory and be assessed again.

#### Data management {19}

EpiData 3.1 software will be used for data entry. To promote the quality and accuracy of data, double data entry and checking will be managed by two independent administrators. The data obtained in this study will be stored in an electronic database. The data administrators and statistician will be the only ones with access to the information. They will also be responsible for data backup. Original case report forms and any other records will be archived at Shanghai University of Sport for a minimum of 5 years from the study end.

#### Confidentiality {27}

The information collected will be used for research purposes and be analysed without the identification of the individuals involved. All the personal information of potential and enrolled participants will not be shared or released. At the end of the trial, all information will be archived at Shanghai University of Sport.

#### Plans for collection, laboratory evaluation and storage of biological specimens for genetic or molecular analysis in this trial/future use {33}

Not applicable because no samples will be collected.

### Statistical methods

#### Statistical methods for primary and secondary outcomes {20a}

The full analysis set includes all randomised participants who take at least one session of intervention. If follow-up data of primary outcomes are missing, last observation carried forward method and multiple imputation method will be carried out to adjust for the missing data and intention-to-treat analysis was utilised.

The statistical analyses, including calculation of the mean and standard deviation, will be performed using SPSS statistical software (version 20.0 for Windows; SPSS, Inc., Chicago, USA). Descriptive statistics will be used separately for every group and for the total sample. Continuous variables will be presented as means, standard deviation, and 95% confidence intervals. And categorical variables will be expressed percentages and absolute numbers. The normality of distribution for quantitative data will be assessed using the Kolmogorov–Smirnov test. Chi-squared tests or Fisher’s exact tests (categorical variables) and one-way ANOVA or nonparametric tests (continuous variables) will be used respectively to test the demographic differences among the IFM, EFM and control groups.

The average effects (difference between the three groups) for all the variables will be estimated using general linear models of multivariate of analysis for repeated measurements (MANOVA) test, which incorporates the three intervention groups (control * IFM * EFM), time (baseline * post-intervention * follow-up) and group-time interaction. To examine the effect size of the treatment, the partial eta squared will be calculated. Bonferroni’s post hoc test will be applied to identify the differences. Once the significant difference of group-time interaction is detected, the one-way repeated measurements will be performed to determine the difference of effect within each group (pre-intervention, post-intervention and follow-up). Statistical significance will be set at 0.05 for all analyses. Analysis will be carried out by an independent researcher.

#### Interim analyses {21b}

Not applicable. Interim testing will not be conducted in this study.

#### Methods for additional analyses (e.g. subgroup analyses) {20b}

Not applicable. No subgroup will be established in this study.

#### Methods in analysis to handle protocol non-adherence and any statistical methods to handle missing data {20c}

An intention-to-treat analysis will be conducted according to the original group allocation. If any participant withdraws from the trial, the last observation carried forward method and multiple imputation method will be respectively carried out to adjust for the missing data.

#### Plans to give access to the full protocol, participant-level data and statistical code {31c}

The datasets analysed during the current study are available from the corresponding author on reasonable request.

### Oversight and monitoring

#### Composition of the coordinating centre and trial steering committee {5d}

The trail steering committee will be formed up of two main investigators in this study, the coordinating centre and the administrative staff of funding. In addition, the group of coordinating centre will be formed up of teachers in Sport Medicine and Rehabilitation Centre, Shanghai University of Sport, and the administrative staffs in community centres.

#### Composition of the data monitoring committee, its role and reporting structure {21a}

The main investigator will email the ethics committee of the University about adverse events or any other changes in the trial. Additionally, after the trial, the main investigator will report the results, adverse event log and publication to the funding committee.

#### Adverse event reporting and harms {22}

The interventions that involve strength training will be performed under the supervision of the qualified physical therapist. Even during the follow-up period, the guidance will be provided weekly for the security of home-based interventions. All participants will be instructed to report any abnormal reactions or uncomfortable feelings experienced to any researcher. Additionally, all adverse events will be recorded truthfully in the trial. The physical therapist and outcome assessors will also collect and record adverse events or any other unintended effects during the interventions and laboratory test, such as musculoskeletal problems (i.e. muscle strain or joint pain). These results will be reported to the main investigator in weekly research meetings.

#### Frequency and plans for auditing trial conduct {23}

The Project Management Group will report the study progress in the form of weekly research meetings. Meanwhile, the Trial Steering Group and Ethics Committee will meet monthly to review conduct throughout the trial period. Due to the low-risk intervention conducted in this study, it is not necessary to make plans to consider a Data Monitoring Committee.

#### Plans for communicating important protocol amendments to relevant parties (e.g. trial participants, ethical committees) {25}

Any important protocol modifications, including the principal investigator, informed consent form, study protocol regarding eligibility criteria, outcomes and analyses, will be reported to all investigators, trial participants, trial registry and ethics committee of the university.

### Dissemination plans {31a}

The results will be reported in conferences or peer-reviewed journals. The results will also be shared with participants, healthcare professionals and the public through lectures or science handbooks.

## Discussion

Falls, as a major public health issue, negatively affect the quality of life, physical function, mental health and social connectedness of older adults, resulting in significant economic burden experienced by themselves, their families, healthcare providers and the healthcare system [41]. As a direct interface between the body and the ground, the human foot contributes to postural stability in most static postures and dynamic activities [13].

Recently, with the development of foot and ankle biomechanics, intrinsic foot muscles have been found to play an active role in foot core stability [15], which is associated with postural stability [19–21]. Kelly et al. [42] assessed the electromyography data of the healthy male participants while performing two quiet standing postures with varying degrees of difficulty (double- and single-leg stances). They found that the activation of the plantar intrinsic foot muscles (the abductor hallucis, flexor digitorum brevis and quadratus plantae muscles) increased with increasing postural demand, and these intrinsic muscles were recruited in a highly coordinated manner to stabilise the foot and maintain balance in the medio-lateral direction. Further research found that the recruitment of these muscles was positively associated with the postural sway of a single-leg stance task [20]. In another biomechanical study, healthy young individuals were instructed to perform an exercise to fatigue the toe and ankle plantar flexors. Their postural control variables were altered after the fatiguing exercises.

Integrating intrinsic-foot-muscle exercises within the lower extremity resistance training, which has been considered as the effective management of fall, is a novel approach. However, this exercise has demonstrated feasibility and effectiveness in the clinical setting among healthy adults and individuals with ankle instability [25, 26]. However, limited research has explored the relationship between intrinsic-foot-muscle function and postural control in older adults. Furthermore, clinical research that investigates the effectiveness of this intervention on postural control in the elderly is lacking.

In conclusion, this protocol will be conducted as a randomised clinical trial and will investigate the efficacy of a 12-week intrinsic foot muscle training combined with lower extremity resistance training on postural stability outcomes in elderly people with fall risk. In this study, we will test the comprehensive outcomes of postural stability during participants walking, static standing and standing under external disturbance. Furthermore, the function of intrinsic foot muscles to support the arch will be evaluated by several quantified tests. The proposed work has the potential to advance the field of elderly fall rehabilitation management by integrating intrinsic-foot-muscle exercise into a lower extremity resistance intervention that will improve foot function and postural stability and ultimately promote balance confidence. The results of this study will determine the effectiveness and provide scientific evidence to establish comprehensive fall prevention intervention.

## Trial status

Protocol: version 2.0; data 31 December 2020.

Date of recruitment began on 1 February 2021.

Date of recruitment will be completed on 31 December 2021.

## Abbreviations

IFM: Intrinsic-foot-muscle exercise
EFM: Extrinsic-foot-muscle exercise
SF: Short-foot
TC: Towel-curl
COP: Centre of pressure
TUG: The Timed Up and Go
ML: Medial-lateral
AP: Anterior-posterior
TL: Total length
SA: Sway area
COG: Center of gravity
SOT: Sensory organisation test
LOS: Limits of stability test
